# Comparative urine proteomic study involving papillary thyroid carcinoma and benign thyroid nodules

**DOI:** 10.3389/fonc.2025.1551247

**Published:** 2025-04-08

**Authors:** Lilong Wei, Rui Xiao, Zhengguang Guo, Pengpeng Wang, Kexin Zhao, Yun Zhou, Wei Sun, Yongtong Cao

**Affiliations:** ^1^ Department of Clinical Laboratory Center, China-Japan Friendship Hospital, Beijing, China; ^2^ Beijing University of Posts and Telecommunications Hospital, Beijing University of Posts and Telecommunications, Beijing, China; ^3^ Core Facility of Instruments, Institute of Basic Medical Sciences, Chinese Academy of Medical Sciences, School of Basic Medicine, Peking Union Medical College, Beijing, China; ^4^ Department of General Surgery & Obesity and Metabolic Disease Center, China-Japan Friendship Hospital, Beijing, China

**Keywords:** urine proteomics, thyroid papillary carcinoma, benign thyroid nodules, urinary disease markers, urine

## Abstract

**Introduction:**

Accurately differentiating benign and malignant lesions is essential for treatment. We aimed to determine differences in urine proteomics between papillary thyroid carcinomas (PTCs) and benign thyroid nodules (BTNs) and identify biomarkers for the differential diagnosis of these diseases.

**Methods:**

We collected 155 specimens. In the discovery group, 30 PTC and 31 BTN specimens were quantitatively compared using liquid chromatography-tandem mass spectrometry (MS). The diagnostic value of each significantly altered protein was calculated in the MS validation comprising 11 PTC and 10 BTN samples. Ultimately, 36 BTN and 37 PTC specimens were used for ELISA validation.

**Results and discussion:**

Overall, 2,479 proteins were used for quantitative analysis. Compared with benign nodules, papillary carcinomas showed significant increases and decreases in the levels of 169 and 27 proteins, respectively. Neck and thyroid tumors were enriched in the disease or function category. More than 100 proteins showed good performance in the area under the receiver operating characteristic curve (>0.8) upon MS validation. Semaphorin-6D showed good performance (AUC = 0.763) in ELISA validation. Urine proteomics is an effective diagnostic tool for distinguishing benign and malignant thyroid diseases. Semaphorin-6D may serve as a disease marker for large-scale validation and use. Additionally, this study identified potential biomarkers that warrant further investigation.

## 1 Introduction

Thyroid nodules (TNs) are common endocrine diseases with high incidence rates. The detection rate in healthy individuals is 20–70%, of which malignant nodules account for 5–15% of the cases (1, 2). Additionally, malignant TNs (thyroid cancer) account for 95.01% of malignant endocrine system tumors (3). Distinguishing benign and malignant TN lesions influences the formulation of treatment plans; therefore, improving the accuracy of benign and malignant TN differentiation is of great importance (4).

Ultrasonography is the most commonly used method for the qualitative diagnosis of TNs. The Thyroid Imaging Reporting and Data System of the American Radiological Society is the standard for grading TNs worldwide. Research has shown that the Thyroid Imaging Reporting and Data System classification has a high sensitivity and negative predictive value for TNs, whereas the positive predictive value for malignant nodules is relatively low (5). Ultrasound-guided fine-needle aspiration cytology is a recognized reference standard for the qualitative diagnosis of TNs (6). However, this examination is invasive and may yield false-negative results for small early nodules. Hence, developing new biomarkers for the differential diagnosis of TNs can effectively enrich diagnostic and treatment methods for patients with clinical TNs.

Urine is the final metabolic product following blood filtration through the glomeruli and its reabsorption, excretion, and secretion in the renal tubules and collecting ducts. Changes in its composition, quantity, and characteristics provide information about the occurrence, development, and prognosis of urinary system diseases and can reflect the overall metabolic status of the body (7, 8). Urine proteomics uses proteomic research techniques to detect various specific biomarkers in urine and has strong feasibility and operability (9). A proteomic study examining glycation in the urine and blood from normal individuals and patients with papillary thyroid cancer found 134 intact N-glycopeptides (10). Pooled urine samples have previously been used to distinguish benign thyroid goiters from papillary thyroid carcinomas (PTCs) using iTRAQ analysis of urinary proteins (11). With the advancement of proteomics technology, the clinical value of urine is increasing, and urine has gradually become one of the main sources for clinical disease marker identification. In this study, we aimed to use urine proteomics to determine differences between malignant and benign TNs and identify biomarkers for the differential diagnosis of these diseases. To our knowledge, this is the first large-scale detection and analysis of individual thyroid specimens using mass spectrometry (MS) to clarify the value of urine proteomics in disease diagnosis and their diagnostic performance as screening biomarkers.

## 2 Methods

### 2.1 Study population

**Figure 1:**
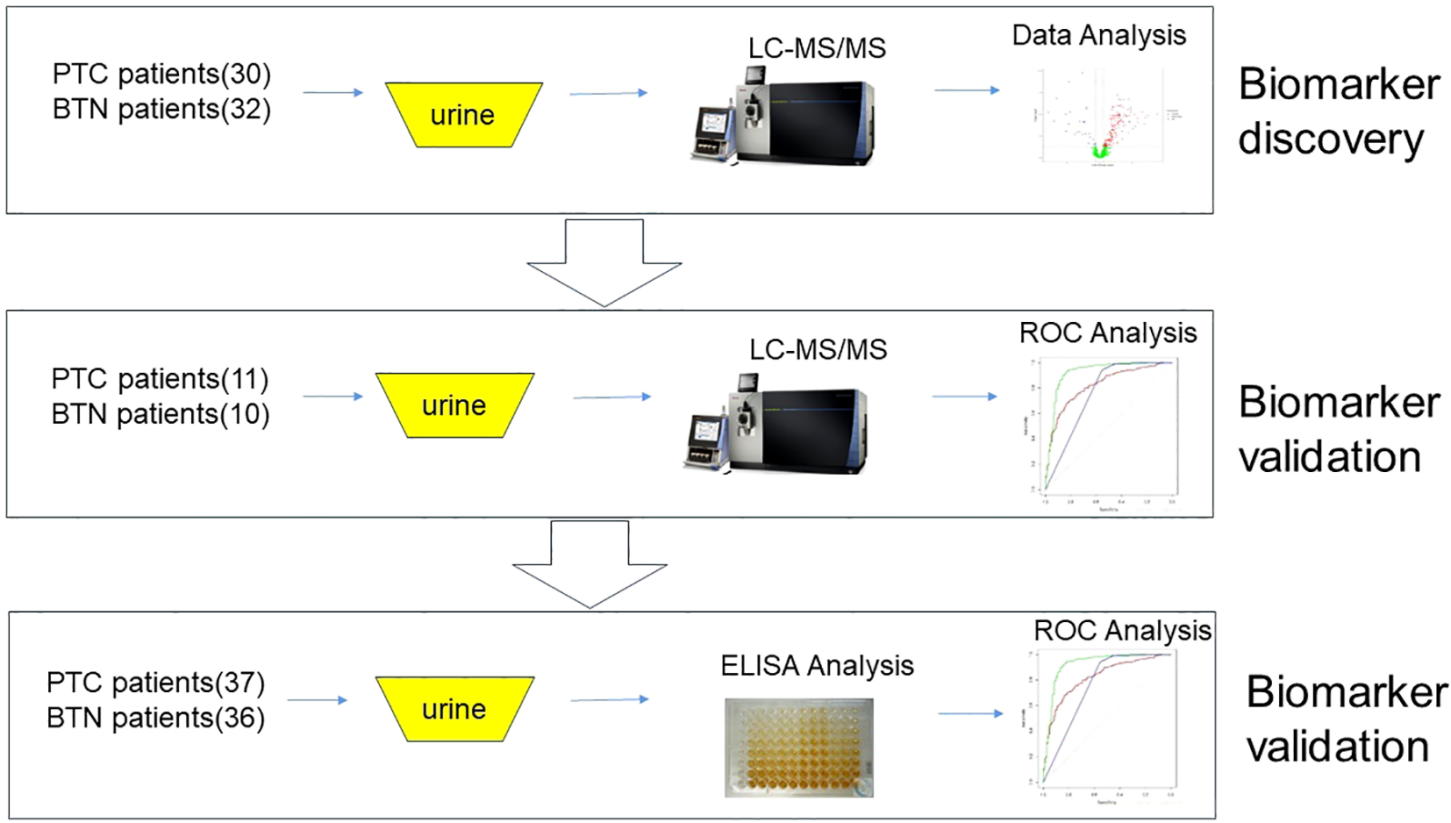
Research design.

The study was approved by the Ethics Committee of China-Japan Friendship Hospital (approval number: 2023-KY-126) and implemented in strict accordance with the relevant ethics standards. During the research process, residual urine samples were collected after clinical testing, which did not increase patient burden and did not involve personal information, and thus the ethics committee approved the application for exemption from informed consent. The inclusion criteria for this study required that patients receive a clear pathological diagnosis. In contrast, patients with previous or concurrent malignant tumors during treatment; urinary tract infections; hematuria; various kidney and urinary system diseases; and liver, kidney, and bone marrow dysfunction were excluded. Details of the patient samples are summarized in **Table 1**; **Supplementary Table S1**. The samples were collected after clinical detection at the China-Japan Friendship Hospital, centrifuged for 10 min at 3000 × *g*, and the supernatant was frozen at -80°C. The experimental design is illustrated in **Figure 1**.

**Table 1:** Basic information about the clinical samples used in this study.

	BTN		PTC	
	Number (M/F)	Age (years)	Number (M/F)	Age (years)
Discovery group	31 (8/23)	48.9 ± 14.1	30 (11/19)	41.8 ± 11.6
Verification group	10 (3/7)	53.7 ± 10.7	11 (5/6)	34.7 ± 14.2
ELISA validation	36 (7/29)	50.1 ± 15.5	37 (12/25)	41.9 ± 13.1

BTN, benign thyroid nodules; PTC, papillary thyroid carcinoma; M/F, male/female; ELISA, enzyme-linked immunosorbent assay.

### 2.2 MS analysis

#### 2.2.1 Sample preparation

The urine samples were precipitated with acetone and subsequently dissolved in a lysis solution containing 8 mol/L urea. The protein concentration in each sample was determined using the Bradford method; proteins were subsequently digested using the filter-aided sample preparation method (12). The details are as follows: Add DTT to a final concentration of 10 mM, incubate at 56°C for 30 minutes to reduce disulfide bonds. Add IAA to a final concentration of 20 mM, incubate at room temperature in the dark for 30 minutes to alkylate cysteine residues. Place the centrifugal filter (MWCO 10-30 kDa) into a centrifuge tube. Load the reduced and alkylated protein sample onto the filter. Centrifuge at 14,000 × g for 15 minutes to remove small molecules (e.g., SDS, DTT, IAA), retain the proteins on the filter. Add 200 µL of urea buffer (8 M urea, 100 mM Tris-HCl, pH 8.5) to the filter. Centrifuge at 14,000 × g for 15 minutes to remove residual SDS and other contaminants. Repeat the above steps 1-2 times to ensure complete buffer exchange. Add 200 µL of ammonium bicarbonate buffer (50 mM NH_4_HCO_3_) to the filter. Centrifuge at 14,000 × g for 15 minutes to exchange the buffer. Repeat the above steps 1-2 times to ensure complete buffer exchange. Add trypsin (enzyme:substrate = 1:50, w/w) to the filter. Incubate at 37°C for 4-16 hours (or overnight) to allow complete protein digestion into peptides. Centrifuge at 14,000 × g for 15 minutes to collect the digested peptides in the flow-through. Add 50 µL of ammonium bicarbonate buffer or water to the filter, and centrifuge to collect residual peptides. Combine the flow-through from multiple centrifugation steps. Desalt the peptide mixture using a C18 solid-phase extraction (SPE) column. Dry the peptides using a vacuum concentrator. The peptide concentration was quantified using the bicinchoninic acid method.

### 2.3 Liquid chromatography-tandem mass spectrometry

For liquid chromatography-tandem mass spectrometry (LC-MS/MS), Orbitrap Exploris 480 (Thermo Scientific, Waltham, MA, USA) was coupled with an EASY nLC 1000 (Thermo Scientific, Waltham, MA, USA) for data-independent acquisition (DAI) MS mode. The elution gradient was 5–30% buffer B2 (0.1% formic acid, 99.9% acetonitrile; flow rate, 0.3 μL/min, peptide elution for 25 min).

For the DIA analysis, a variable isolation window containing 60 windows was used for MS acquisition. Based on the precursor m/z distribution of the merged samples, the number of precursor ions was equal in each separation window. The full scan range was set to 350–1,200 m/z and filtered at a resolution of 120,000, followed by a DIA scan with a resolution of 30,000 (high-energy C-well dissociation collision energy: 30%; automatic gain control target: 200%; maximum injection time: 50 ms).

### 2.4 Data processing

The original DIA data were analyzed using the default settings in Spectraut Pulsar 17.1 (Biognosys, Zurich, Switzerland). Briefly, the retention time prediction type was set as the dynamic indexed retention time. Interference correction was performed at the MS2 level. Peptide strength was calculated by summing the peak areas of the respective fragment ions of MS2, and protein strength was calculated by summing the strengths of each peptide. Cross-run normalization was enabled to correct system differences in LC-MS/MS performance using local normalization strategies. Standardization was based on the assumption that a similar number of peptides are upregulated and downregulated on average and that most peptides in the sample are not regulated throughout the entire run or retention time. Protein inference was performed using the ID selector algorithm implemented in the Spectraut software. All results were filtered through a Q-value cutoff of 0.01 (corresponding to a 1% false discovery rate).

### 2.5 Statistical analysis

Missing values of proteomic data in the samples were independently imputed into different subgroups using the sequential k nearest-neighbor method. Student’s *t*-tests and one-way analyses of variance were used for the statistical analyses of all quantitative data. Statistical significance was defined as a fold change > 1.5 times, *P*<0.05.

### 2.6 Bioinformatics analysis

Hierarchical clustering analysis and volcano plots of differentially expressed proteins were generated using the online data analysis program of the Wukong Cloud platform (https://www.omicsolution.com/wkomics/main). Pattern recognition analyses (principal component analysis and orthogonal partial least squares discriminant analysis) were performed using SIMCA 14.0 (Umetrics, Umeaa, Sweden) software. All differentially expressed proteins between the two groups were analyzed using ingenuity pathway analysis (IPA) software (QIAGEN, Germantown, MD, USA). Categories for disease and function and canonical pathways for the proteins were analyzed and ranked according to their *P*-values. The enrichment significance of each function was calculated using one-sided Fisher’s exact tests.

Receiver operating characteristic (ROC) curves were generated for each candidate biomarker using MetaboAnalyst (http://www.metaboanalyst.ca). A combination ROC analysis was performed using the linear support vector machine algorithm, and the area under the ROC curve (AUC) was calculated.

### 2.7 Enzyme-linked immunosorbent assay validation

Four proteins in urine were validated using enzyme-linked immunosorbent assays (ELISAs). The human semaphorin-6D ELISA kit (Catalogue number EH409RB, Thermo Scientific, Waltham, MA, USA), human matrilin-2 ELISA kit (Catalogue number EH313RB, Thermo Scientific, Waltham, MA, USA), C-X-C motif chemokine 14 ELISA kit (Catalogue number ELH-CXCL14, RayBiotech, Norcross, GA, USA), and mucin-13 ELISA kit (Catalogue number NBP2-76698, Novus, St. Louis, MO, USA) were used according to the manufacturer’s instructions.

## 3 Results

### 3.1 Urinary protein identification by LC-MS/MS

**Figure 2:**
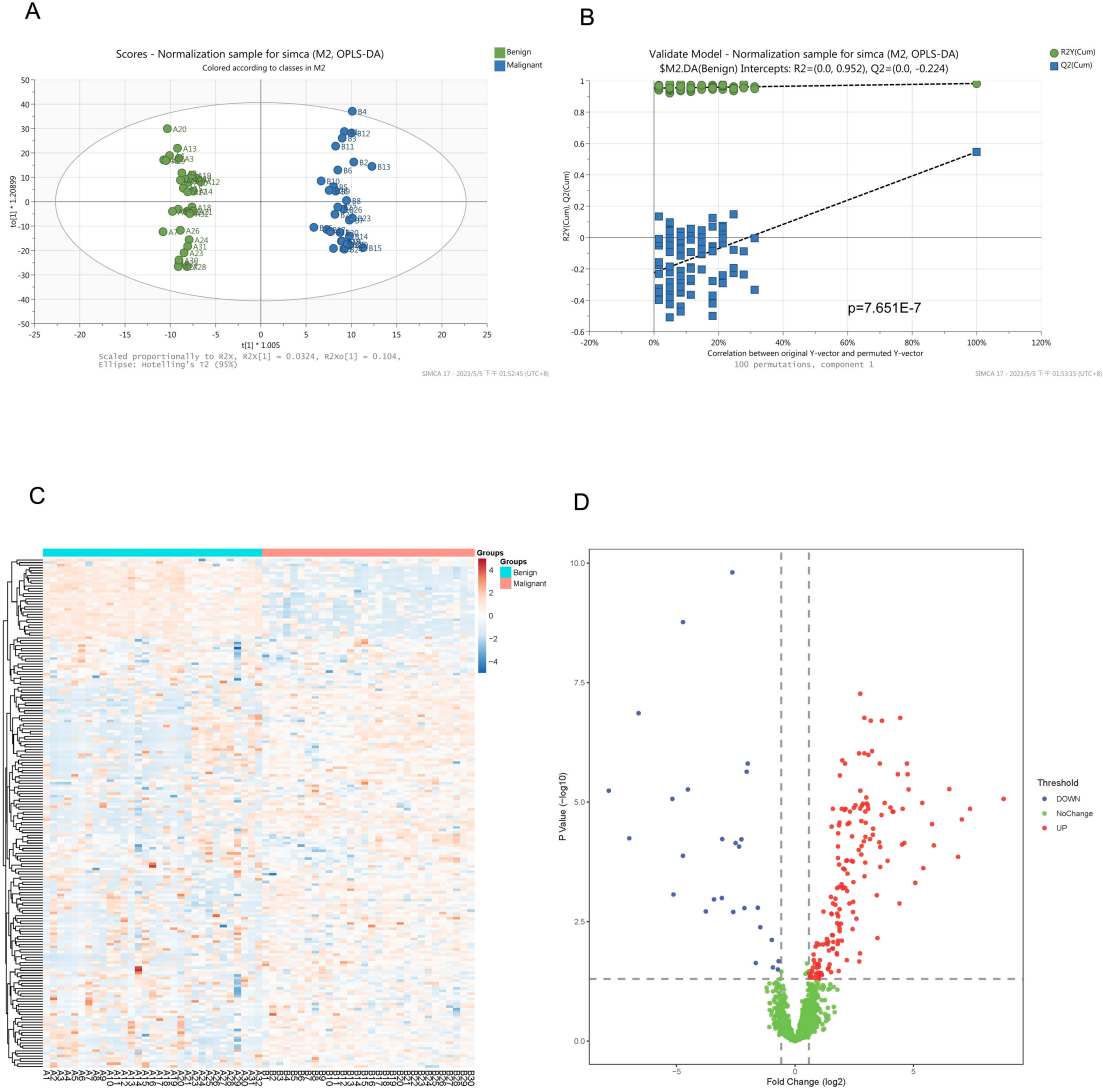
Visual display of the proteins identified by mass spectrometry including significantly differential proteins. **(A)** An orthogonal partial least squares discriminant analysis (OPLS-DA) of proteins identified by mass spectrometry. **(B)** Permutation analysis (Validate Model) for the proteins identified by mass spectrometry. **(C)** Heatmap of the significantly different proteins between the two groups. **(D)** Volcano plots of the differential proteins between the two groups.

In our study, 86 urine samples were analyzed using LC-MS/MS. A total of 3842 proteins were identified that exhibited at least one unique peptide, and 2479 proteins were quantitatively analyzed. This analysis yielded quantitative information in more than half of the quality control samples (detailed information is provided in the **Supplementary Documents, Sheet 1**). For instances of missing proteomic data in the samples, the sequential k nearest-neighbor method was used to independently input different subgroups. A total of 196 proteins were found to be significantly altered by a fold change > 1.5 times (*P* < 0.05; detailed information is provided in the **Supplementary Documents, Sheet 2**). Visual analysis of the proteins identified by MS in benign and malignant TNs and significantly differentially expressed proteins was conducted (**Figure 2**).

### 3.2 Function analysis of significantly changed proteins by IPA

**Figure 3:**
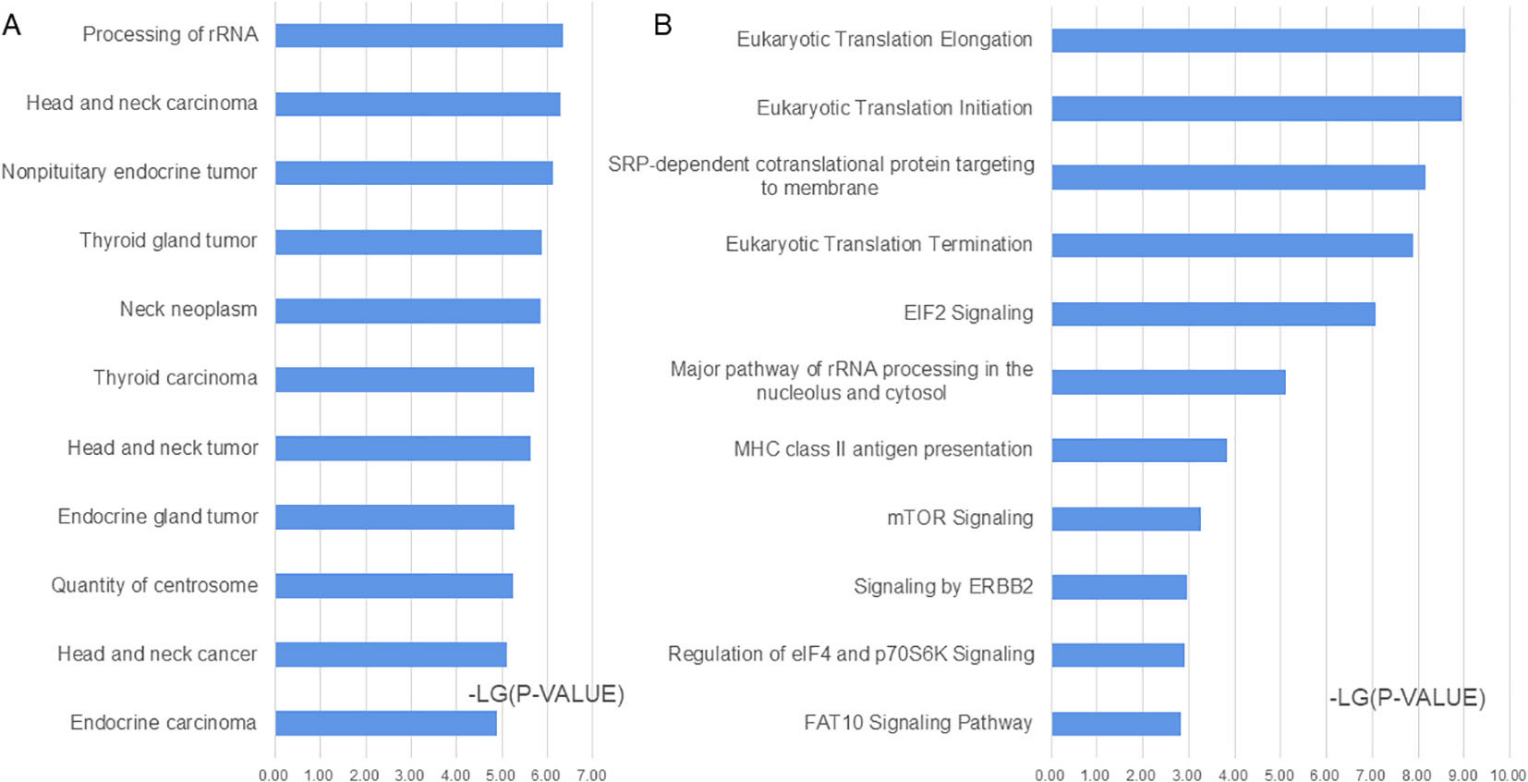
Functional analysis of the significantly changed proteins according to ingenuity pathway analysis (IPA). **(A)** Disease and biofunction analysis enriched by IPA. **(B)** Canonical pathway analysis enriched by IPA.

A functional analysis was conducted on 196 significantly differentially expressed proteins using the IPA software. In the disease and biofunction analyses, proteins associated with head and neck carcinoma, non-pituitary endocrine tumor, thyroid gland tumor, neck neoplasm, epithelial thyroid cancer, head and neck neoplasia, and endocrine gland tumor were enriched (**Figure 3A**; **Supplementary Documents, Sheet 5**). In the canonical pathway analysis, eukaryotic translation, EIF2 signaling, mTOR signaling, ERBB2 signaling, regulation of EIF4 and p70S6K signaling, FAT10 signaling pathway, and MHC class II antigen presentation were enriched (**Figure 3B**; **Supplementary Documents, Sheet 5**).

### 3.3 Discovery and verification of protein markers

To determine the diagnostic value of significantly different proteins in benign and malignant TNs, MS validation was performed on different samples. To evaluate the stability of the MS detection system and reduce the bias of MS detection, we conducted MS analysis on quality control samples every 15 samples. The quality control sample consisted of a mixture of the experimental and other urine samples. Principal component analysis and correlation analyses were conducted on the quality control samples, and the quality control correlation was 0.975–0.985 (**Supplementary Figure S1**).

ROC analysis of significantly different proteins in both the discovery and validation groups was performed using MetaboAnalyst software. Based on the AUC, 100 proteins exhibited good performance (AUC > 0.8) (**Supplementary Data**), suggesting that these biomarkers can be used to diagnose PTC. Based on the identification level of urinary proteins in the discovery group and relevant literature, we conducted an in-depth analysis of four proteins using ROC analysis and ELISA validation.

**Figure 4:**
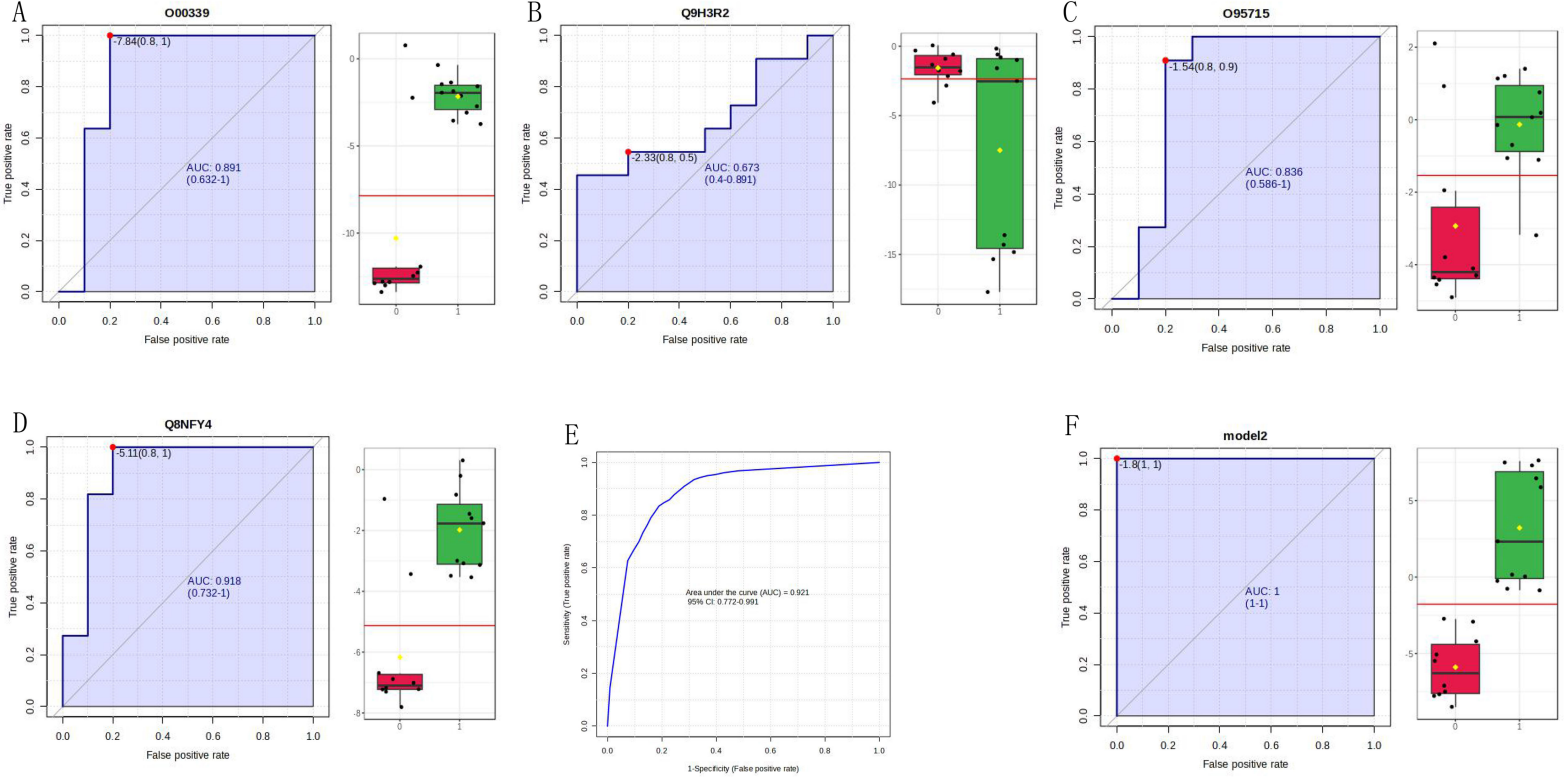
Validation of four proteins by mass spectrometry (MS). **(A)** Receiver operating characteristic (ROC) analysis for matrilin-2 (ID: O00339). **(B)** ROC analysis for mucin-13 (MUC13, ID: Q9H3R2). **(C)** ROC analysis for C-X-C motif chemokine 14 (ID: O95715). **(D)** ROC analysis for semaphorin-6D (ID: Q8NFY4). **(E)** 10-fold cross-validation in the discovery group of the four-protein panel. **(F)** ROC analysis for the four-protein panel.

**Figure 5:**
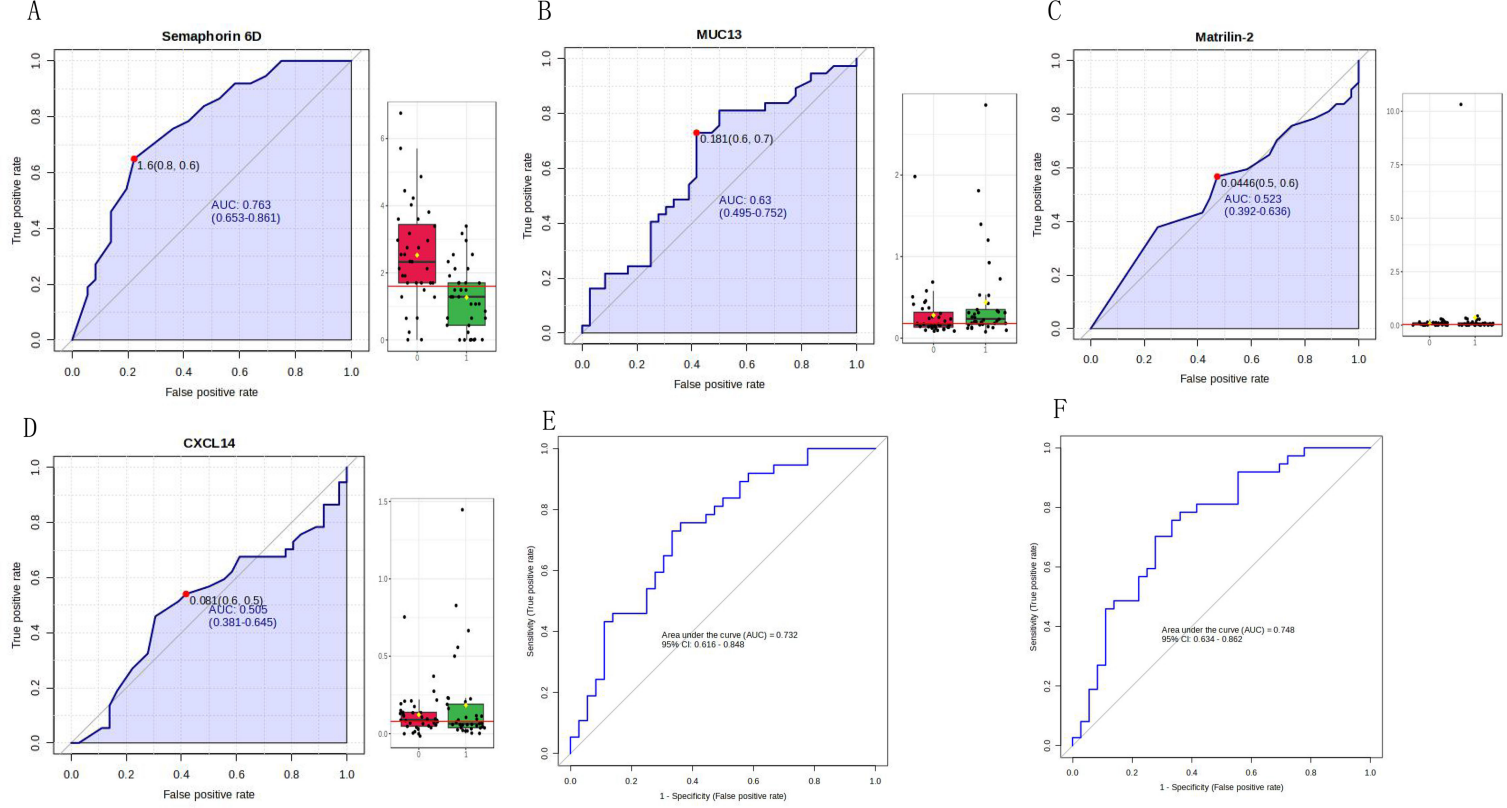
Validation of four proteins by enzyme-linked immunosorbent assay (ELISA). **(A)** Receiver operating characteristic (ROC) analysis for semaphorin-6D (ID: Q8NFY4). **(B)** ROC analysis for mucin-13 (MUC13, ID: Q9H3R2). **(C)** ROC analysis for matrilin-2 (ID: O00339). **(D)** ROC analysis for C-X-C motif chemokine 14 (ID: O95715). **(E)** 10-fold cross-validation of the four-protein panel. **(F)** 10-fold cross-validation of the panel consisting of semaphorin-6D and mucin-13.

The four identified proteins were semaphorin-6D (ID: Q8NFY4), C-X-C motif chemokine 14 (ID: O95715), mucin-13 (MUC13, ID: Q9H3R2), and matrilin-2 (ID: O00339). The ROC analysis of each protein in the MS validation is shown in **Figure 4**. The four-protein panel significantly improved the diagnostic accuracy of the individual markers with AUC values of 0.921 (95% confidence interval, 0.772–99; **Figure 4E**) and 1.0 (95% confidence interval, 1–1; **Figure 4F**) in the discovery and validation groups, respectively. Validation of these four proteins by ELISA was performed simultaneously in 73 clinical samples. Semaphorin-6D showed good performance (AUC = 0.763), while the other proteins showed lower performance (**Figure 5**). Combining these proteins as a panel did not increase the diagnostic performance (**Figures 5E, F**).

## 4 Discussion

Disease markers related to thyroid cancer have been studied, mainly including tissues and body fluids. The expression profiles of the galectin gene family, especially galectin-1 and galectin-3, were found to differ between primary and metastatic papillary thyroid carcinoma in tissue samples of benign and malignant thyroid nodules (13); Based on the proteome of thyroid tissue samples, metastasis related markers were recorded (14, 15). Plasma proteomic studies have found that LCAT, GPX3, and leukotriene b4 can serve as potential biomarkers for thyroid cancer (16), and serum ISG15 and PLXNB2 can also serve as potential biomarkers (17). A study on urine proteomics found differential proteins in the differentiation between lung cancer and thyroid cancer (18). There were differential proteins between the urine proteome and glycoproteome, but the sample size is only 15 cases and there is a lack of validation for the relevant proteins (10). Given the unique advantages of *in vitro* detection of urine samples, we conducted in-depth research on the identification markers of benign and malignant thyroid nodules using a large sample size.

In the present study, urinary proteins of 86 urine samples from benign and malignant TNs were identified using proteomic technology. A total of 3842 proteins were identified that exhibited at least one unique peptide, and 2479 proteins were quantitatively analyzed. 196 proteins were found to be significantly altered by a fold change > 1.5 times (*P* < 0.05). The significantly altered protein number was the highest in current researches. Compared with previous studies, significantly differential proteins including galectin-3, which was identified in tissues (13), PRTN3 and GGT1 have been reported in urine proteomic studies (10). However, most of the differentially expressed proteins were not the same, which may be related to sample type, sample size, or depth of proteomic identification.

In our study, the results revealed significant differences in urine proteomics between benign and malignant TNs. In **Figures 2A, B**, the urine proteomic data of the experimental and validation groups can be clearly distinguished based on the orthogonal partial least squares discriminant analysis. Heatmap and volcano plots showing the significantly different proteins between the two groups display the differences between these two sets of specimens. These findings demonstrate the potential of urine proteomics for distinguishing between benign and malignant TNs.

To better understand the differences between benign and malignant TNs, functional analysis was performed using IPA software to determine significantly different proteins. Based on the disease and biofunction analyses, the most significantly enriched functions were related to neck or thyroid gland tumors, and more than 150 proteins were related to thyroid carcinoma (**Supplementary Data**). In the canonical pathway analysis, many pathways related to translation and signaling were enriched. Eukaryotic translation initiation factors promote tumor development (19), and EIF3A was identified as a significantly different protein in this study, which has been reported to delay tumor cell growth following gene knockdown (20). The EIF2 signaling pathway is related to many kinds of tumors (21, 22). The mTOR signaling pathway regulates many major cellular processes, is associated with a growing number of pathological conditions, including cancer (23), and could be a therapeutic target for thyroid cancer (24). ERBB2 could drive somatic mutations in Chinese PTC tumors (25), and ERBB4, a member of the ERBB2 signaling pathway, was significantly altered, which is reported to be related to poor prognosis and malignant development in patients with PTC (26). The signal recognition particle-dependent co-translational proteins targeting the membrane pathway are also related to trophoblastic tumors and lung carcinoma (27, 28). This pathway is speculated to be related to PTC.

To analyze whether the differentially expressed proteins discovered in the discovery group could distinguish between benign and malignant TNs, the identified proteins were validated using both MS and ELISA. Based on MS validation, many proteins showed good performance, suggesting that differential protein biomarkers have the potential to distinguish between benign and malignant TNs using MS. However, the validation sample size in this study was relatively small. Nevertheless, these data provide new clues about the diseases and a basis for future studies.

Semaphorin-6D, C-X-C motif chemokine 14, mucin-13, and matrilin-2 were selected for ELISA validation. These four proteins changed significantly in both the discovery and validation groups and had AUC values ranging from 0.673 to 0.918 individually, with an AUC value of 1 when using the four-protein panel. Semaphorin-6D is involved in cell development and migration as a guiding factor in head and neck, lung, and breast cancers (29–31). Some subtypes of the chemokine C-X-C motif are associated with thyroid carcinoma (32–34). Mucin-13 high-molecular-weight transmembrane glycoprotein is frequently and aberrantly expressed in a variety of epithelial carcinomas, including gastric, colorectal, and ovarian cancers (35, 36). Mucin-13 signaling is thought to be activated in PTC. In addition, matrilin-2 expression can improve the diagnosis of cytologically indeterminate thyroid cancers (37). However, the diagnostic value of these four proteins has not been previously reported, especially in the urine. Notably, the AUC value was lower for ELISA validation than for MS validation. In our experiment, the results of MS validation were excellent, but owing to the small sample size for MS validation, larger-scale validation is still needed. Semaphorin-6D performed well, whereas the performance of the other three proteins was not ideal. The sample detection concentrations of C-X-C motif chemokine 14 and matrilin-2 were very low, and the reagent kit used in the assay may be unsuitable for urine sample detection. Moreover, owing to limited research funding, only four proteins were validated by ELISA; additional candidate markers must be identified for further validation.

## 5 Conclusion

In summary, urinary proteomics can be used to effectively distinguish between benign and malignant TNs. Utilizing MS, many protein biomarkers were identified that could differentiate these two diseases. Finally, our findings support that semaphorin-6D can be assayed by ELISA to improve PTC diagnosis.

## Data Availability

The datasets presented in this study can be found in online repositories. The names of the repository/repositories and accession number(s) can be found in the article/**Supplementary Material**.
