# Supplementary figures and images for: Comparative urine proteomic study involving papillary thyroid carcinoma and benign thyroid nodules
Source: Front Oncol. 2025 Apr 8;15:1551247. doi: 10.3389/fonc.2025.1551247 (PMC12011787; doi:10.3389/fonc.2025.1551247)

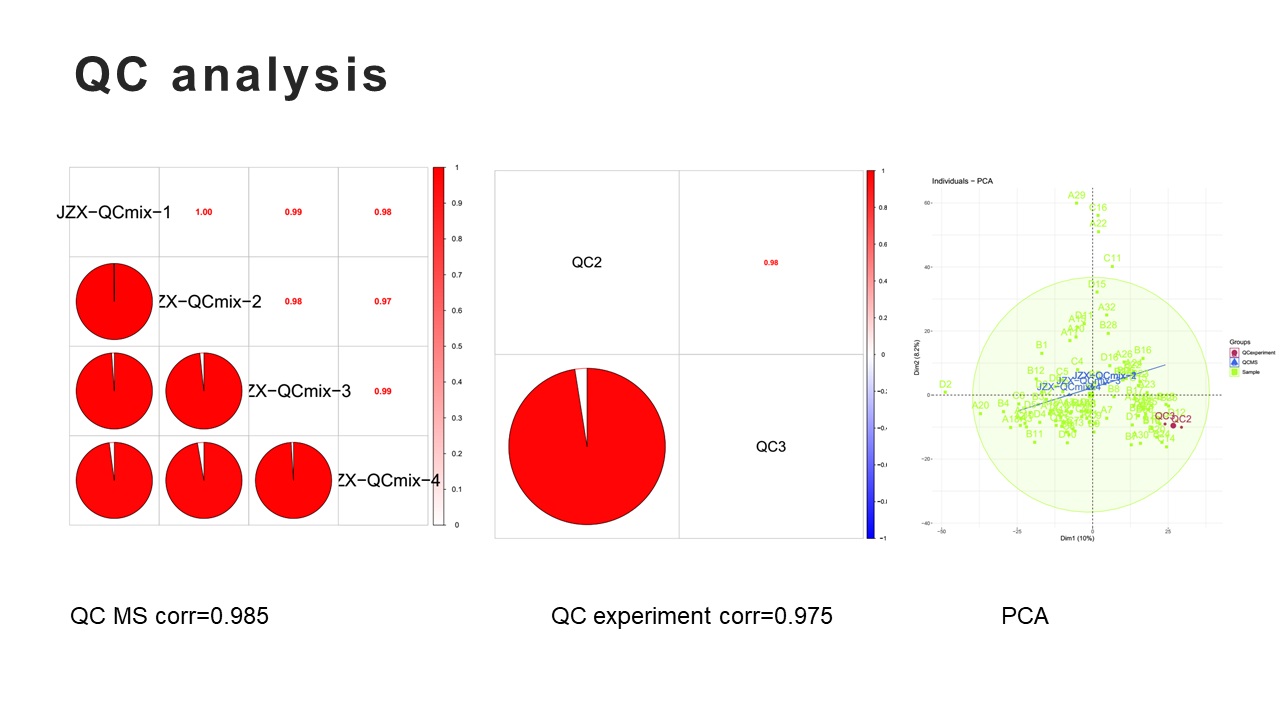

Supplement: Supplementary file 1 [file Image1.jpeg]
